# Relationship between skeletal muscle mass and glycemic parameters in individuals with young-onset type 2 diabetes mellitus

**DOI:** 10.1007/s11845-025-04111-2

**Published:** 2025-10-07

**Authors:** Anupama Harihar, Sahana Shetty, Shivashankar K. N, Shyamasunder Bhat N, Dhiren Punja, Sachin Kumar, G. Arun Maiya

**Affiliations:** 1 Centre for Podiatry & Diabetic Foot Care and Research, Department of Physiotherapy, Manipal College of Health Professions, Manipal Academy of Higher Education, Madhav Nagar, Manipal, 576104 Karnataka India; 2 Department of Endocrinology, Kasturba Medical College, Manipal, Manipal Academy of Higher Education, Manipal, Karnataka India; 3 Department of Medicine, Kasturba Medical College, Manipal, Manipal Academy of Higher Education, Manipal, Karnataka India; 4 Department of Orthopaedics, Kasturba Medical College, Manipal, Manipal Academy of Higher Education, Manipal, Karnataka India; 5 Department of Physiology, Kasturba Medical College, Manipal, Manipal Academy of Higher Education, Manipal, Karnataka India

**Keywords:** Adult-onset diabetes, Bioelectric impedance, Body composition, Early-onset diabetes, Predictive value, Skeletal muscle mass

## Abstract

**Background:**

Young-onset type 2 diabetes mellitus (T2DM) is an increasingly prevalent condition characterized by rapid progression. Apart from adipose tissue, there has been growing attention to the relationship between T2DM and total body skeletal muscle mass (SMM).

**Aim:**

This study investigated the relationships between body composition indicators and glycemic parameters in young-onset T2DM patients versus young, healthy adults, aiming to identify predictive markers with optimal cutoff values for the early identification of young-onset T2DM.

**Methods:**

A cross-sectional study was conducted among 252 participants aged 18–40 years, including 96 young-onset T2DM patients and 156 non-T2DM individuals. Glycemic parameters and body composition variables were assessed via bioelectrical impedance analysis. Statistical analysis included correlation, multiple logistic regression, and receiver operating characteristic curve analysis to determine optimal SMM% cutoff values for young-onset T2DM prediction.

**Results:**

The SMM% displayed a significant negative correlation with HbA1c (*p* =  − 0.624) and FBG (*p* =  − 0.656). VF demonstrated a positive correlation with both HbA1c (*p* = 0.636) and FBG (*p* = 0.580). Logistic regression analysis identified SMM, VF, and subcutaneous fat as significant predictors of HbA1c levels. Receiver operating characteristic analysis revealed gender-specific SMM% cutoff values of 23.14% for females and 28.6% for males, with high sensitivity and specificity.

**Conclusion:**

Reduced SMM% and increased VF are significant predictors of young-onset T2DM. The study established the optimal gender-specific cutoff value of SMM% for identifying individuals at risk of young-onset T2DM in the Indian population. Incorporating body composition assessments into clinical practice may facilitate early detection and targeted interventions.

## Introduction

 Diabetes mellitus, a well-known metabolic condition, results in defective blood glucose regulation, causing insufficiency in maintaining regular blood glucose levels [1]. People who are diagnosed with type 2 diabetes mellitus (T2DM) prior to the age of 40 years are classified as having young-onset T2DM [2, 3]. T2DM accounts for over 90% of overall diabetes cases [4]. Among these, young-onset diabetes has become increasingly prevalent. The prevalence of young-onset T2DM is rising in various mid- and high economic status countries; the magnitude of this increase seems to be more substantial among those aged 15 to 39 years and is more pronounced in Asian countries [5]. In the case of young-onset T2DM, it is linked to a more aggressive phenotype and a rapid advancement of complications early in life [6]. The increasing incidence of T2DM is associated with various risk factors such as an aging population, economic growth, urban development, and obesity stemming from a lack of physical activity and excessive consumption [7, 8]. These lifestyle behaviors lead to excessive visceral fat accumulation, releasing proinflammatory factors, and causing an imbalance in endocrine functions. Eventually, these factors may all merge, resulting in insulin resistance and T2DM development in the young adult population [9].

Earlier research indicated that body composition measurements like body mass index, waist-to-hip ratio, body fat mass, muscle mass, and particularly visceral fat are linked to a relatively high prevalence rate and are recognized as independent risk factors for T2DM. Notably, the waist-to-hip ratio and visceral fat are identified as predictive indicators of T2DM in younger adults [10, 11]. Insulin resistance in the liver is associated with visceral fat, whereas insulin resistance in the periphery is related to total body fat and subcutaneous fat [12, 13]. These changes serve as key pathogeneses in the onset and progression of T2DM [14]. Hence, quantitative assessment of body adiposity is important. The pattern of fat distribution and the presence of significant muscle mass serve as both risk factors and possible causes of the onset of T2DM at a relatively young age [15]. Assessing body composition can allow prompt and efficient detection of risk factors for T2DM, aiding in its early recognition and prevention.

Recently, in addition to adipose tissue, there has been growing attention to the relationship between T2DM and total body skeletal muscle mass (SMM). A study by Waddell et al. suggested that SMM was significantly lower in T2DM patients compared to non-T2DM patients [16]. SMM represents a significant indicator of body composition evaluation and is essential for the storage and metabolism of glucose [17]. These findings suggest that SMM may be an independent indicator of the regulation of blood glucose [18].

The identification of T2DM or the prediabetic stage depends on the concentrations of glycated hemoglobin (HbA1c) and fasting blood glucose according to the standardized guidelines [19]. However, the influence of body composition variables on glycemic parameters is essential to assess. Hence, this research aimed to explore the relationships between body composition indicators and glycemic parameters in young-onset T2DM patients versus young, healthy adults in India. Additionally, we aimed to establish the optimal cutoff value for the percentage of total body skeletal muscle mass for the prediction of young-onset T2DM. The findings of this research may serve as a reference cutoff value for screening for diabetes in the young adult population. Indeed, primary prevention strategies and enhanced research in the diabetes field are offered.

## Methods

### Study design

The current study has a cross-sectional (observational) research design that was carried out and documented in accordance with the “STROBE—Strengthening the Reporting of Observational Studies in Epidemiology guidelines” [20].

### Study setting

This research was conducted between May 2024 and March 2025 at a tertiary hospital in India. Institutional and ethical board approvals (IEC1: 81/2023) were obtained before the commencement of the study, and the study was registered under the Clinical Trial Registry of India (CTRI/2023/08/056051 dated 02/08/2023). A convenient sampling method was employed to recruit the sample size. Consent in writing was obtained from the participants before their participation. The participants were screened from the medical and endocrinology outpatient departments of a tertiary care hospital. The included participants were composed of a population of similar ethnicities, races, and cultural habits to control for potential confounding variability.

### Sample size

This study encompasses a total of 252 participants. The sample size was calculated considering the prevalence (*P* = 17.2%) [21] and Z-score corresponding to the desired confidence level (1.96 for 95% confidence) into the standard calculation formula, *n* = (Z^2 × P × (1−P))/E^2.

### Participants

A selection criterion was established to minimize the confounding factors. A total of 252 participants out of the 275 screened met the selection criteria and were included in the study. The study included individuals aged between 18 and 40 years of either gender, and also individuals who were diagnosed with young-onset T2DM in the past 3 months by a physician or endocrinologist. However, individuals should not be on any drugs/medications for glycemic control (for lifestyle and dietary modification only). The exclusion criteria were as follows: (a) people diagnosed with latent autoimmune diabetes in adults, type 1 diabetes, pancreatic diabetes, steroid-induced diabetes, gestational diabetes, or prediabetes; (b) pregnant or postpartum women within 12 weeks were excluded as well due to the possibility of impaired glucose tolerance during pregnancy; (c) patients with a history of any chronic condition, such as parathyroid disease, metabolic syndrome, or kidney/liver/heart disease; and (d) individuals receiving any hormonal or ongoing steroid therapy.

### Variable measurement

#### Measurement of blood glucose

Blood glucose levels were assessed via a conventional glucometer in all the included participants. The diagnostic criteria for diabetes followed the “11th edition of the International Diabetes Federation diabetes atlas,” 2025, which indicates a random blood glucose measurement greater than 220 mg/dl, a fasting blood glucose measurement more than 126 mg/dl, or a blood glucose level 2 h after consuming a 75-g oral glucose tolerance test that is higher than 200 mg/dl [22, 23]. Therefore, fulfilling any of the three following criteria under a uniform pattern by a solitary trained examiner was noted. Based on these criteria, individuals with young-onset T2DM and non-T2DM were grouped. Individuals with a history of previously diagnosed T2DM were verified through their medical records.

### Measurement of body composition

A standard calibrated handheld body impedance analyzer (BIA) machine was used to measure the participant’s body composition. This method has been validated for its accuracy and reproducibility [24, 25]. The digital, portable, non-invasive device, “The Omron Karada Scan (HBF-702 T Body Fat Analyzer, Omron Health Care Pvt Ltd, China),” utilizes the tetra-polar BIA method for its operation. This device sends an electrical current of 500 μA at a frequency of 5 kHz to evaluate the entire body by measuring impedance through contact electrodes [26]. Participants’ age, gender, and height using a stadiometer were entered manually into the device via a stadiometer. The participant was then asked to stand at a footpad and hold the handles with their arms at a 90-degree angle to obtain regional body composition data. The following measures were taken to avoid compromising the accuracy of the procedure: (a) we conducted the measurements in the morning to prevent dehydration; (b) participants were instructed to avoid eating and drinking caffeinated beverages or alcohol, and refrained from smoking; and (c) they were also instructed not to engage in any intense physical activities for at least 2 hour before the body composition assessment [27]. The same evaluator conducted the test for all participants to avoid any measurement bias. The body composition variables included were body weight, body mass index (BMI), total fat body percentage (TF%), total skeletal muscle mass percentage (SMM%), subcutaneous fat percentage (SCF%), visceral fat level (VF), resting metabolic rate (RMR), muscle mass percentage, and subcutaneous fat percentage in the arms, trunk, and legs.

### Bias

To reduce measurement bias, a single trained investigator performed all body composition assessments to ensure consistency across participants. Blood glucose measurements were conducted using standardized laboratory protocols by a certified technician.

### Statistical analysis

Statistical evaluation was conducted using the Jamovi software version 2.3.28. Descriptive statistics were calculated to find the mean values and standard deviations of continuous variables. Normality tests were conducted to identify variations in descriptive statistics between participants with young-onset T2DM and those without T2DM (Shapiro–Wilk *p* < 0.05). There were no missing data for any of the variables included in the analysis. To understand the relationship, Spearman’s correlation matrix analysis was implemented between glycemic and body composition variables. To evaluate the difference in body composition variables between individuals with young-onset T2DM and those without T2DM, an independent *t*-test analysis was conducted. Furthermore, multiple logistic regression analysis of body composition variables was performed to identify predictors for young-onset T2DM. The ROC, receiver operating characteristic curve, along with the AUC, area under the curve, was employed to determine the best cutoff values of SMM% for forecasting T2DM in the young adult population. The significance level was established at *p* ≤ 0.05.

## Results

### Demographic details

The study involved 252 participants in total, with an average age of 30.95 ± 9.65 years. This included 143 (56.7%) male and 109 (43.2%) female individuals. Among these, 156 (61.9%) were healthy non-T2DM participants, and 96 (38%) were diagnosed with young-onset T2DM. The mean age of the non-T2DM participants was 28.3 ± 5.86 years, whereas the young-onset T2DM participants had a mean age of 33.68 ± 5.41 years. The mean fasting blood glucose (FBG) level was 80.15 ± 13.25 mg/dL in the non-T2DM group and 151.15 ± 52.53 mg/dL in the young-onset T2DM group. Similarly, the mean HbA1c level was 4.88 ± 0.39% in non-T2DM participants and 8.21 ± 1.53% in young-onset T2DM participants. Differences between the non-T2DM and young-onset T2DM groups were analyzed using the Mann–Whitney *U* test, as presented in Table 1. There was found to be no significant difference among some of the body composition variables, namely TBF%, muscle mass% in trunk and legs, and subcutaneous fat% in arms and legs (*P* ≥ 0.05) between the groups; hence, less skewed data were implemented among all included participants.

**Table 1:** Comparison of age, glycemic parameters, and body composition variables between non-T2DM and young-onset T2DM individuals

Parameter	Non-T2DM (*N* = 156)	Young-onset T2DM (*N* = 96)	*p* value
Age	28.3 ± 5.86	33.6 ± 5.40	< 0.001
Glycated hemoglobin (HbA1c)	4.8 ± 0.38	8.2 ± 1.52	< 0.001
Fasting blood glucose	80.1 ± 13.24	151.1 ± 52.53	< 0.001
Body mass index	23.6 ± 3.54	28.73 ± 4.49	< 0.001
Total body fat%	29.7 ± 4.13	33.2 ± 5.66	0.055
Skeletal muscle mass%	36.4 ± 5.20	25.14 ± 4.39	0.002
Subcutaneous fat %	23.5 ± 4.40	29.6 ± 5.39	< 0.001
Visceral fat level	7.6 ± 2.88	14.6 ± 3.47	< 0.001
Muscle mass (Arm)%	31.8 ± 6.24	28.8 ± 5.45	0.004
Muscle mass (Trunk)%	23.8 ± 5.87	22.2 ± 6.85	0.143
Muscle mass (Leg)%	36.5 ± 6.60	33.5 ± 6.60	0.089
Subcutaneous fat (Arm)%	28.7 ± 9.52	34.1 ± 9.58	0.07
Subcutaneous fat (Trunk)%	22.2 ± 7.35	27.9 ± 6.09	0.049
Subcutaneous fat (Leg)%	30.88 ± 6.85	36.02 ± 7.95	0.056

### Associations of body composition variables with glycemic parameters

Spearman correlation analysis, depicted in Table 2, revealed significant associations between body composition variables and glycemic parameters (FBG and HbA1c levels). The SMM% displayed a significant negative correlation with HbA1c (*P* = −0.624) and FBG (*P *= −0.656). On the contrary, VF demonstrated a positive correlation with both HbA1c (*P* = 0.636) and FBG (*P* = 0.580). SCF% and TFP% were also positively correlated with HbA1c and FBG, although the correlations were weaker than those for VF.

**Table 2:** Correlations between body composition variables and glycemic parameters

Glycemic parameters	Body mass index	Total body fat%	Skeletal muscle mass%	Subcutaneous fat%	Visceral fat
HbA1c (r value)	0.436	0.302	− 0.624	0.535	0.636
Fasting blood glucose (*r* value)	0.462	0.334	− 0.656	0.421	0.58

Values represent Spearman’s correlation coefficients (r) with corresponding two-tailed *p*-values. Negative r values indicate inverse correlations, whereas positive *r* values indicate direct correlations. *p*-value for all the variables was < 0.001

The statistical analysis comparing body composition variables such as BMI, TBF%, SMM%, SCF%, and VF levels among the young-onset T2DM and non-T2DM groups is shown in Table 3. All body composition variables showed significant differences between the groups (*P* < 0.001). However, the effect sizes vary, with the most substantial differences observed in SMM% and VF levels, indicating distinct patterns of body composition are associated with diabetes status.

**Table 3:** Multiple linear regression of body composition variables for the prediction of young-onset T2DM

Predictor	Estimate	SE	95% confidence interval		t	*p*	VIF	Tolerance
			Lower	Upper				
Intercept	6.3318	1.3861	3.5924	9.0711	4.568	<.001	1.91	0.524
Body mass index	− 0.0231	0.0342	− 0.0907	0.0445	− 0.675	0.501	1.39	0.720
Total body fat%	0.0186	0.0266	− 0.034	0.0712	0.699	0.486	1.71	0.583
Skeletal muscle mass%	− 0.0972	0.0208	− 0.1382	− 0.0561	− 4.678	<.001	1.47	0.679
Subcutaneous fat%	0.0682	0.0248	0.0193	0.1172	2.756	0.007	2.31	0.432
Visceral fat	0.125	0.038	0.0499	0.2	3.29	0.001	1.91	0.524

The predictors include body mass index (BMI), total body fat percentage, skeletal muscle mass percentage, subcutaneous fat percentage, and visceral fat. Estimates represent regression coefficients, with standard errors (SE), 95% confidence intervals (lower and upper bounds), t-statistics (t), and *p*-values (p). Variance inflation factor (VIF) and tolerance values are included to assess multicollinearity among predictors. A *p*-value < 0.05 indicates statistical significance

### Variables related to body composition for predicting T2DM

Multiple linear regression analysis was conducted for the body composition parameters, namely BMI, TBF%, SMM%, SCF%, and VF level. The analysis identified that SMM%, VF, and SCF% were significant predictors of HbA1c levels. SMM% had a negative association with HbA1c (*β* = −0.0972, *P* < 0.001), while VF (*β* = 0.1250, *P* = 0.001) and SCF% (*β* = 0.0682, *P* = 0.007) were positively associated (Table 3). The model accounted for 49% of the variability in HbA1c levels (R^2^ = 0.490, adjusted R^2^ = 0.473, *P *< 0.001).

### Diagnostic cutoff for skeletal muscle mass percentage in predicting young-onset T2DM

**Fig. 1:**
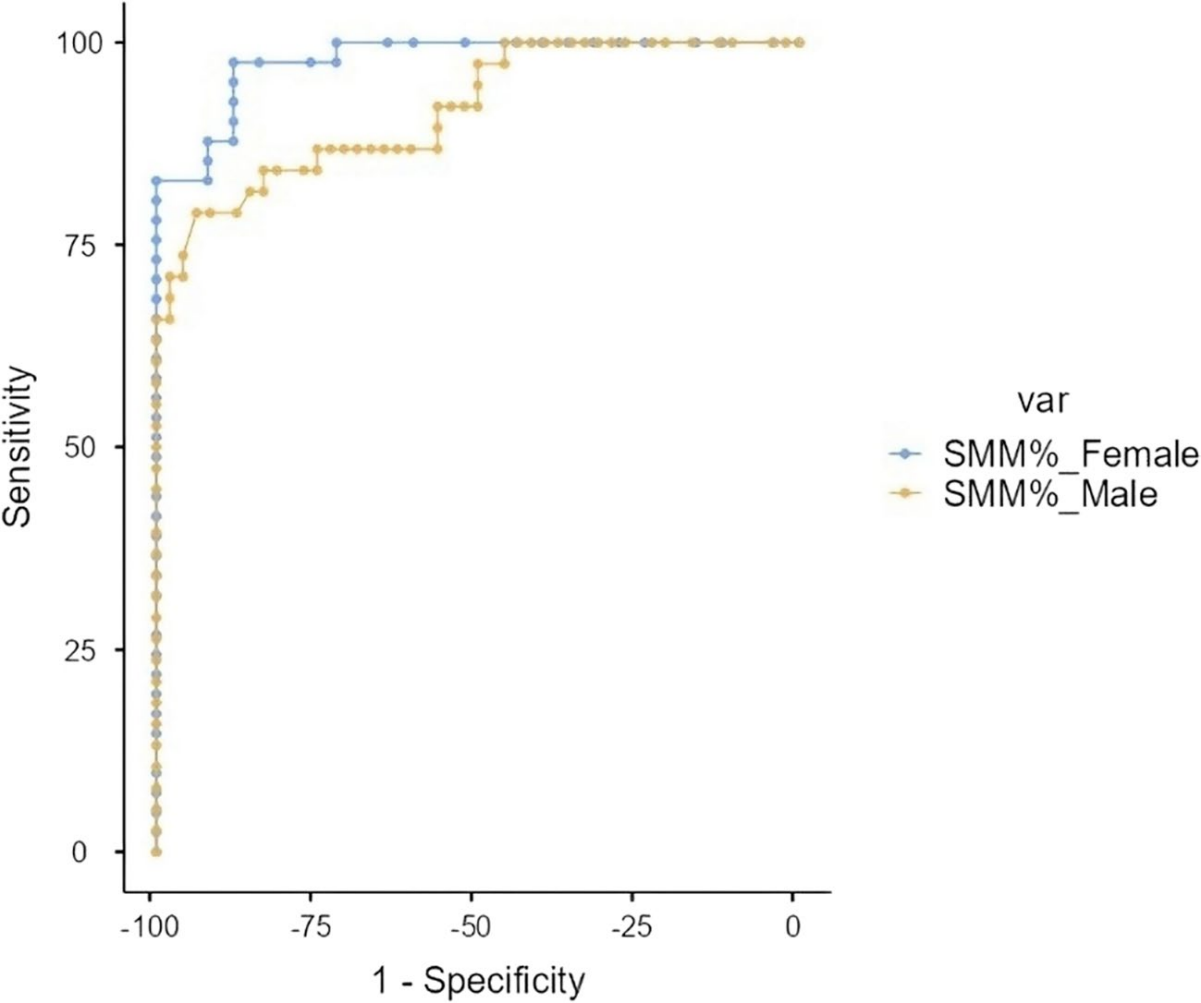
ROC curve of Skeletal Muscle Mass Percentage of females and males as a predictor of young-onset T2DM

To identify the best possible cutoff value for SMM% in predicting young-onset T2DM, a ROC curve analysis was conducted. For females, a cutoff value of 23.14% was established, achieving a sensitivity of 97.56% and a specificity of 88%. In contrast, a cutoff percentage of 28.6% was identified for males, yielding a sensitivity of 78.95% with a specificity of 93.75%, along with an AUC of 0.917 (Table 4) (depicted in Fig. 1). The Youden’s index for this cutoff was 0.727, indicating a robust diagnostic performance. This suggests that SMM% is a reliable marker for identifying individuals at risk of young-onset T2DM.

**Table 4:** Diagnostic cutoff for skeletal muscle mass percentage in predicting young-onset T2DM

Characteristic	Cutoff value	Sensitivity (%)	Specificity (%)	Positive predictive value (%)	Negative predictive value (%)	Youden’s index	Area under the curve
Skeletal muscle mass% (females)	23.14	97.56%	88%	93.02%	95.65%	0.856	0.978
Skeletal muscle mass% (males)	28.6	78.95%	93.75%	90.91%	84.91%	0.727	0.917

## Discussion

The present cross-sectional observational study evaluated the relationship between body composition indicators and glycemic parameters in young-onset T2DM patients versus young, healthy adults. The study revealed that SMM% was a significant predictor for young-onset T2DM, with a gender-specific cutoff value of 23.14% in females and 28.6% in males among the Indian population. The glycemic parameters were markedly elevated in the young-onset T2DM group, which was consistent with the diagnostic criteria for diabetes and the metabolic dysregulation associated with this condition. Hence, evaluating how body composition affects the diagnosis and prognosis of diabetes is crucial. These findings may aid in the need for targeted interventions in young adult populations to address the rising prevalence of T2DM.

## Relationships between body composition indicators and glycemic parameters

The study’s correlation analysis revealed significant associations between BMI, SMM%, TBF%, SCF%, and VF levels, and FBG and HbA1c. VF exhibited a strong positive correlation with these indices, highlighting its role as a major contributor to hyperglycemia, insulin resistance, inflammation, and dyslipidemia. VF is considered hazardous compared with SCF, as it is more metabolically active and associated with metabolic syndrome and cardiovascular diseases [28]. Several previous studies have demonstrated that VF, or adiposity, is a reliable marker for assessing the risk of T2DM [29–31]. Our study strongly validates the association of VF in individuals with young-onset T2DM. The possible reason is due to the increased blood flow in visceral adipose tissue, which responds to norepinephrine being greater, and there is an increase in sympathetic nervous system activity, while the effectiveness of insulin in suppressing lipolysis is reduced [32].

SCF% and TBF% also correlated positively with the glycemic index, although they had weaker associations than VF. Recent studies have contradicted that SCF, particularly in the lower body, is associated with a more favorable metabolic profile. It is linked to enhanced insulin sensitivity and a lower risk of impaired glucose tolerance [33, 34]. This may be due to the hormonal profile of SCFs compared to VFs, which may contribute to their protective effects [35]. The risk of developing T2DM increases with a higher TBF. In a Korean cohort, the risk for T2DM significantly increased for men with TBF% over an optimal cutoff value of 22.8% and for women with TBF% over 32.9% [36]. Hence, our study findings align with those of previous literature emphasizing the differential impact of fat distribution on metabolic health.

## Skeletal muscle mass as a predictor of young-onset T2DM

Skeletal muscle is the body’s most significant insulin-sensitive tissue, and changes in muscle mass can greatly influence glucose metabolism [37]. The present study shows the negative association between SMM% and HbA1c. It implies that lower SMM is linked to a higher risk of young-onset T2DM, hence reinforcing the protective role of skeletal muscle in glucose metabolism. Moreover, low muscle mass results in a reduced capacity for glucose disposal, and increased mitochondrial dysfunction leads to subsequent insulin resistance, contributing to the risk of T2DM [38]. Additionally, excessive lipid storage in the muscle cells activates novel protein kinase C, which disrupts insulin-stimulated glucose signaling and transportation [39]. The ROC curve analysis demonstrated the diagnostic potential of SMM% in predicting young-onset T2DM. Evidence also indicates that higher SMM (adjusted by BMI) is associated with a lower risk of T2DM in both genders [37, 40]. In comparison, a study by Hong et al. among a healthy population with an approximate average age of 38 years found that the greater the likelihood of the development of young-onset T2DM in the presence of less muscle mass [41]. Hence, interestingly, in individuals younger than 50 years, there is a stronger association between relative muscle mass and the development of diabetes [41]. On the contrary, few studies have indicated a lack of association between SMM and diabetes [42], which may be attributed to variations in the age demographics of the participants (> 50 years).

The occurrence of decreased muscle mass is higher in males than in females with T2DM [43]. The identified cutoff values for SMM% provided high sensitivity and specificity, particularly in females, with an optimal cutoff value of 23.14% and an AUC of 0.917, indicating an excellent diagnostic marker, and in males with a cutoff value of 28.6%. The cutoff values obtained are marginally lower than the normal SMM ranges, which are 25.9–27.9% for females and 32.9–35.7% for males in healthy individuals [27]. Reduced muscle mass is notably linked to greater variations in glucose levels in males, whereas this association is not observed in females [43]. These findings indicate that muscle mass plays a more critical role in glucose stability in men with T2DM. The relationship between fat mass and muscle mass influences cardiovascular risk factors in different ways for men and women. In women, a combination of low muscle mass and low fat mass correlates with elevated HbA1c levels, which is an indicator of cardiovascular disease [44].

## Strengths, limitations, and future recommendations

The strength of our study depicts a consistent relationship between body composition and glycemic parameters, but the independent correlation and cutoff values for SMM% in individuals with young-onset T2DM among the Indian population have been established for the first time. However, the fact that most of the participants were recruited from a single hospital’s outpatient department is a limitation of this study, which may lead to unavoidable selection bias. Additionally, other potential predictors, such as genetic history or socioeconomic status, were not eliminated during screening. This can provide scope for future research focusing on the impact of reduced skeletal muscle mass on lipid profiles to evaluate the likelihood of cardiovascular diseases in people with young-onset T2DM. Additionally, anthropometric methods are simple but subject to validation, and alternative measures of body composition analysis, such as DEXA or MRI, can be expensive and not feasible. Hence, BIA can be a strong recommendation for routine screening in clinical setups.

## Conclusion

The findings of this study revealed that the relationship of body composition variables such as SMM, VF, and SCF indicators is strongly associated with glycemic parameters among healthy individuals versus individuals with young-onset T2DM. A reduced SMM% and increased VF are significant predictors of young-onset T2DM. Also, importantly, the study established the optimal gender-specific cutoff value of SMM% for identifying individuals at risk of young-onset T2DM in the Indian population. Hence, the findings emphasize the importance of incorporating body composition assessments into routine clinical practice for the early diagnosis and prevention of diabetes in young adults.

## Data Availability

In view of the confidentiality and privacy of the participants, the datasheets and other supporting documents are available from the corresponding author (Dr. G Arun Maiya: arun.maiya@manipal.edu) upon reasonable request.
